# Network Analysis of Dysregulated Immune Response to COVID-19 mRNA Vaccination in Hemodialysis Patients

**DOI:** 10.3390/vaccines12101146

**Published:** 2024-10-07

**Authors:** Yi-Shin Chang, Jessica M. Lee, Kai Huang, Christen L. Vagts, Christian Ascoli, Russell Edafetanure-Ibeh, Yue Huang, Ruth A. Cherian, Nandini Sarup, Samantha R. Warpecha, Sunghyun Hwang, Rhea Goel, Benjamin A. Turturice, Cody Schott, Montserrat H. Martinez, Patricia W. Finn, David L. Perkins

**Affiliations:** 1 Department of Medicine, University of Illinois at Chicago, Chicago, IL 60612, USAjlee835@uic.edu (J.M.L.); khuang49@uic.edu (K.H.); cvagts2@uic.edu (C.L.V.); cascoli@uic.edu (C.A.); sam.warpecha@gmail.com (S.R.W.); bturtu2@uic.edu (B.A.T.); mmart344@uic.edu (M.H.M.); davidperkins@salud.unm.edu (D.L.P.); 2 Department of Bioengineering, University of Illinois at Chicago, Chicago, IL 60612, USA; 3 Department of Microbiology and Immunology, University of Illinois at Chicago, Chicago, IL 60612, USA; 4 Department of Medicine, Stanford University, Palo Alto, CA 94305, USA; 5 Department of Medicine, University of Colorado Denver, Aurora, CO 80045, USA; 6 Department of Medicine, University of New Mexico, Albuquerque, NM 87131, USA

**Keywords:** gene regulatory network, transcriptomics, epigenetics, hemodialysis, end-stage renal disease

## Abstract

Introduction: End-stage renal disease (ESRD) results in immune dysfunction that is characterized by both systemic inflammation and immune incompetence, leading to impaired responses to vaccination. Methods: To unravel the complex regulatory immune interplay in ESRD, we performed the network-based transcriptomic profiling of ESRD patients on maintenance hemodialysis (HD) and matched healthy controls (HCs) who received the two-dose regimen of the COVID-19 mRNA vaccine BNT162b2. Results: Co-expression networks based on blood transcription modules (BTMs) of genes differentially expressed between the HD and HC groups revealed co-expression patterns that were highly similar between the two groups but weaker in magnitude in the HD compared to HC subjects. These networks also showed weakened coregulation between BTMs within the dendritic cell (DC) family as well as with other BTM families involved with innate immunity. The gene regulatory networks of the most enriched BTMs, likewise, highlighted weakened targeting by transcription factors of key genes implicated in DC, natural killer (NK) cell, and T cell activation and function. The computational deconvolution of immune cell populations further bolstered these findings with discrepant proportions of conventional DC subtypes, NK T cells, and CD8+ T cells in HD subjects relative to HCs. Conclusion: Altogether, our results indicate that constitutive inflammation in ESRD compromises the activation of DCs and NK cells, and, ultimately, their mediation of downstream lymphocytes, leading to a delayed but intact immune response to mRNA vaccination.

## 1. Introduction

End-stage renal disease (ESRD), the most advanced stage of chronic kidney disease (CKD), is highly prevalent in the United States: the United States Renal Data System reports over 2219 per million population and 135,972 new cases in 2021 alone. Of these, 83.8% underwent in-center hemodialysis (HD); nonetheless, ESRD patients on HD faced a mortality rate of 191.5 per 1000 person-years, with infections and cardiovascular disease as the most common causes [1]. ESRD manifests as a duality of immune incompetence driven by uremic toxicity, which leads to increased risk of infection, co-existing with immune activation driven by the accumulation of proinflammatory cytokines, which contributes to the progression of atherosclerotic lesions and vascular disease [1,2]. The uremic state in ESRD is known to contribute to the chronic activation of natural killer (NK) cells, monocytes, dendritic cells (DCs), T cells, and B cells and the production of proinflammatory cytokines that predisposes these cells to anergy, apoptosis, and overall premature immunological aging that could be traced to epigenetic remodeling [3]. As a result, this duality has not only led to higher susceptibility to infection, but also lower response to vaccination: higher vaccination failure rates have been reported for hepatitis B virus, influenza virus, *Clostridium tetani*, and *Corynebacterium diphtheriae* [4,5].

More recently, studies of the COVID-19 BTN162b2 mRNA vaccine in HD patients presented a more optimistic outlook compared to responses to prior vaccines, demonstrating seroconversion rates as high as 96%, though there was still some reduction in SARS-CoV-2 IgG antibodies in HD patients [6,7,8,9,10]. The improved immune protection has been attributed to the novel design philosophy of mRNA vaccines, which engages both innate and adaptive components of the immune response through local inflammation at the site of injection as well as replicating key features of natural viral infection, including the production of antigenic proteins by host cells and the induction of type I interferon (IFN) and downstream Th1 polarization, without the same risks that live vaccines would pose to immunocompromised populations [11]. At the same time, our group previously reported delayed transitioning from the innate immune response to the adaptive immune response in HD patients compared to healthy controls (HCs) over the course of the two-dose BTN162b2 vaccination regimen, likely as a product of chronic immune activation/desensitization in this patient population [10]. Work by other groups has also demonstrated a decreased innate immune response to SARS-CoV-2 glycoprotein stimulation in HD patients compared to HCs at 4–5 and 8–9 weeks post-vaccination [12]. Further complicating these matters are conflicting reports of both innate and adaptive immune mechanisms underlying ESRD [13,14,15,16].

While transcriptomic approaches have tackled this complexity by extracting key differentially expressed genes associated with immune phenotypes, there is growing recognition for examining the regulatory processes that govern gene expression as well. Biological networks offer a holistic characterization of the complex dysregulated interactions among genes and regulators in ESRD [17]. At the most fundamental level, these networks consist of nodes, which typically represent genes or other biological targets of interest, connected to other nodes by edges, which can represent co-expression or other types of relationships of relevance [18]. When these relationships can be quantified, e.g., as correlation coefficients, network analysis can aid in pinpointing the most significant relationships from background noise. Therefore, we propose to investigate mechanisms of regulatory dysfunction underlying the delayed immune response to COVID-19 (BNT162b2) mRNA vaccination in HD subjects as compared to healthy controls (HCs) at the (1) transcriptional, (2) epigenetic, and (3) cellular levels by means of network analysis centered around (1) gene module co-expression and (2) gene transcriptional regulation, and combined with (3) immune cell populations computationally deconvoluted from expression data (Figure S1).

## 2. Results

### 2.1. Demographic and Clinical Characterization of Hemodialysis and Control Cohorts

We utilized previously published bulk RNA-seq data from our group, which recruited 20 ESRD patients (estimated glomerular filtration rate < 15 mL/min) on maintenance hemodialysis (HD) and 20 matched healthy control (HC) cohorts who received the original two-dose BNT162b2 mRNA vaccine series against SARS-CoV-2 in 2021 [10]. Peripheral blood transcriptomes were profiled at four timepoints: baseline before (V1D0) and seven days after the first dose (V1D7), and baseline before (V2D0) and seven days after the second vaccine dose (V2D7). Only subjects with all four time points represented were included in this study for network construction, resulting in a final total of 12 HD subjects and 19 HC subjects. Demographics and clinical data for this subset is shown in Table 1. Aside from mean age and race, for which Black/African American subjects were more dominantly represented in the HD cohort, and White/Caucasians more dominantly represented in the HC cohort, the two cohorts shared similar demographics. Common ESRD comorbidities including type 2 diabetes mellitus and hypertension were more highly represented in the HD cohort. On average, HD patients had serum creatinine of 8.8 ± 2.7 (normal range: 0.50–1.50 mg/dL for men and 0.40–1.20 mg/dL for women) and underwent 44 ± 37 months on dialysis before the dialysis session on their first round of vaccination.

### 2.2. Differential Gene Expression Analysis Shows Delayed Onset of Vaccine-Induced Innate Immunity in HD

As described in Chang et al., differential gene expression analysis between pre- and post-vaccination time points (V1D0 vs. V1D7, V2D0 vs. V2D7), followed by gene set enrichment analysis using blood transcription modules (BTMs), revealed temporal differences in BTMs between the HC and HD cohorts [10]. In HC subjects, T cell activity was increased and monocyte activity decreased at V1D7 relative to V1D0, while plasma cell activity was increased at V2D7 relative to V2D0, illustrating an expected transition from innate to cell-mediated immunity beginning with the first vaccine dose. In contrast, HD subjects featured increased myeloid cell activity at V1D7 and increased PLK signaling at V2D7. As we have previously shown comparable antibody responses by V2D7 in both groups, the persistence of innate immune activity well past the first dose in HD patients suggests that the immune response may be delayed compared to HCs, potentially through aberrant transcriptional regulation.

### 2.3. BTM Co-Expression Networks Show Globally Weakened Coregulation of Vaccine-Induced Immune Response in HD Subjects

First, co-expression networks were constructed based on the first principal component, or eigengene, of the BTMs for each group. As described previously, BTMs represent 334 modules of co-expressed genes, established based on human blood transcriptomes from over 30,000 human blood transcriptome samples across more than 500 studies [19]. The dimensional reduction from thousands of genes to several hundred BTMs enables a high-level understanding of the regulatory patterns of the vaccine-induced immune response in HD versus HC subjects. It further enables the downstream construction of single-subject networks with a higher signal-to-noise ratio.

**Figure 1:**
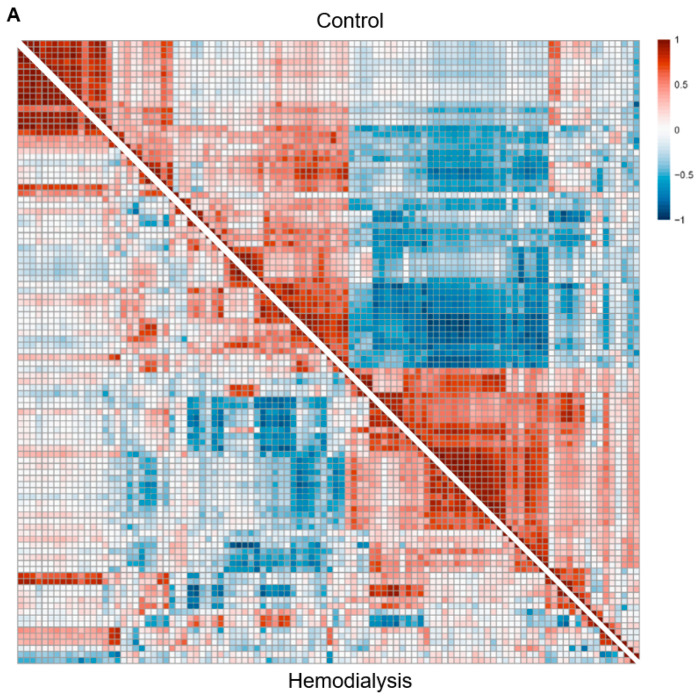

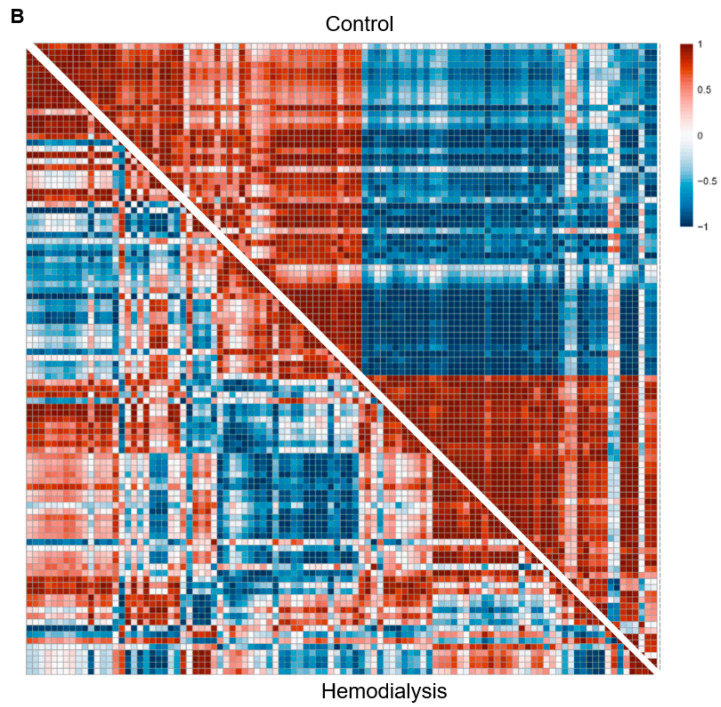
Blood transcription module (BTM) co-expression networks for hemodialysis (HD) group demonstrate similar but weaker patterns of co-expression compared to healthy control (HC) networks. (**A**) Group-level BTM co-expression networks are constructed from pairwise Pearson correlations between pairs of BTM eigengenes across subjects and time points before each vaccination dose (V1D0, V2D0) and one week after each vaccination dose (V1D7, V2D7), separately for HC (top diagonal) and HD (bottom diagonal) groups. (**B**) Representative single-subject BTM co-expression networks for HC (top diagonal) and HD (bottom diagonal) groups are likewise constructed across time points for each subject. Color scale characterizes these correlations as positive (red), negative (blue), or no correlation (white), with darker colors indicating stronger correlation.

Co-expression could be characterized as positive or negative, where positive co-expression indicates that the activation of one component corresponds with the activation of the other, and negative co-expression when the activation of one component corresponds with repression of the other. Both the HD and HC BTM co-expression networks demonstrated overall similar co-expression patterns, represented as network edges, with 91% of the 3449 statistically significant edges in the across-group network (*p* < 0.05, FDR-adjusted) sharing the same direction of coregulation for both groups (1653 positive; 1496 negative) (Figure 1A). However, the HD participants generally exhibited weaker edge strength, or coregulation, between BTMs compared to the HCs. Of the edges that were positively coregulated in both groups, 241 (15%) exhibited weaker regulation in the HD group compared to HCs (*p* < 0.05, FDR-adjusted), while only 90 (5%) exhibited stronger regulation (Figure 1A). Similarly, of the edges that were negatively coregulated in both groups, 236 (16%) edges exhibited weaker negative regulation in HD participants (*p* < 0.05, FDR-adjusted), while only 21 (1%) exhibited stronger negative regulation (Figure 1A). These group-level BTM co-expression patterns were similarly observed in networks constructed at the single-subject level for each group, indicating that weakened coregulation is a consistent feature across individual HD subjects (Figure 1B). 

**Figure 2:**
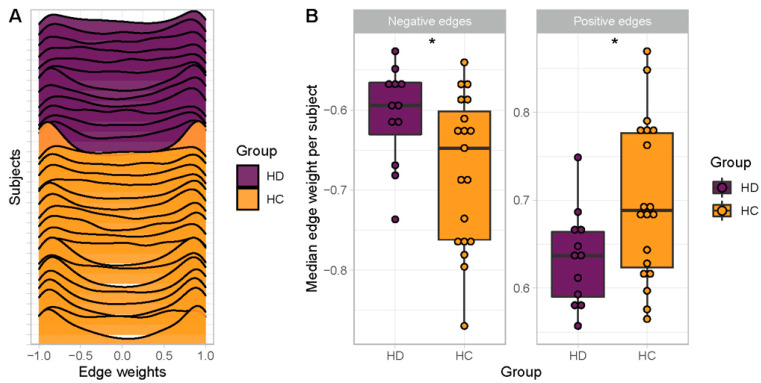
Single-subject BTM co-expression networks demonstrate weaker positive and negative co-expression in HD subjects compared to HCs. (**A**) Density plots of edge weights per subject in HC (yellow) compared to HD (purple) subjects. (**B**) Box-and-whisker plots of median edge weight across significantly positively co-expressed edges (right) and across significantly negatively co-expressed edges (left), separately per subject. * *p* < 0.05.

When visualizing co-expression strength as edge weight distributions across the entire network per subject, the HC subjects exhibited a bimodal distribution with peaks at highly positive and negative edges that is largely lost in the HD subjects (Figure 2A). This comparison is confirmed statistically with the median edge weight (Fisher Z-value) across all positively coregulated edges being significantly less positive in the HD subjects compared to HCs (*p* < 0.05) (Figure 2B). Similarly, the median edge weight across all negatively coregulated edges was significantly less negative in the HD subjects compared to HCs (*p* < 0.05) (Figure 2B). These comparisons maintained statistical significance when incorporating SARS-CoV-2 as a covariate. Overall, the BTM co-expression networks at both the group and single-subject levels demonstrate weakened positive and negative coregulation of the immune response in HD subjects over the course of the two-dose BNT162b2 mRNA vaccine regimen.

### 2.4. BTMs Involved in Innate Immunity Demonstrate Less Positively Correlated Co-Expression in HD Subjects

**Figure 3:**
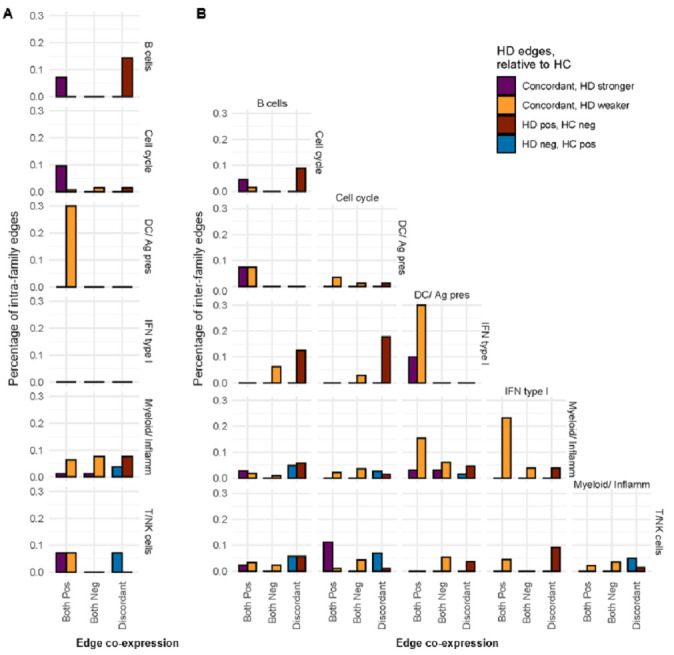
Comparison of group-level BTM family associations between HC and HD groups show weaker positive co-expression in HD. Co-expression patterns for both HC and HD groups are shown between each set of BTM families (cell cycle, DC/Ag pres, IFN type I, Myeloid/Inflamm, T/NK cells) based on percentage of (**A**) intrafamily edges and (**B**) interfamily edges that were positively co-expressed (both pos), negatively co-expressed (both neg), or co-expressed with opposite signs (discordant). Edge co-expression was classified as concordantly stronger in HD (purple), concordantly weaker in HD (yellow), or discordant with positive (brown) or negative (blue) edges in HD subjects. Percentages of edges with FDR-corrected *p* < 0.05 were considered significantly different between HC and HD groups.

In order to identify the key immune processes with compromised coregulation in HD subjects, the BTMs were further categorized into six broader families (B cells, cell cycle, dendritic cell/antigen presentation [DC/APC], type I interferon [IFN type I], myeloid activity/inflammation [Myeloid/Inflamm], and T/NK cells), and member edges from HD or HC co-expression networks were then correlated within the same family (intrafamily edges) as well as between different families (interfamily edges) [20]. Notably, the DC/APC family showed the most altered intrafamily co-expression, with 30% of intrafamily edges demonstrating weakened positive co-expression in HD subjects relative to HCs (Figure 3A). Of these, the most weakened edge was between LI.M43.0 (Myeloid, dendritic cell activation via NfkB (I)) and LI.S5 (DC surface signature). This indicates that many of the BTMs within the DC/APC family are less strongly co-activated with one another in HD. There was also substantial weakening of positive interfamily co-expression pairwise between the DC/APC, IFN type I, and Myeloid/Inflamm families (Figure 3B).

**Figure 4:**
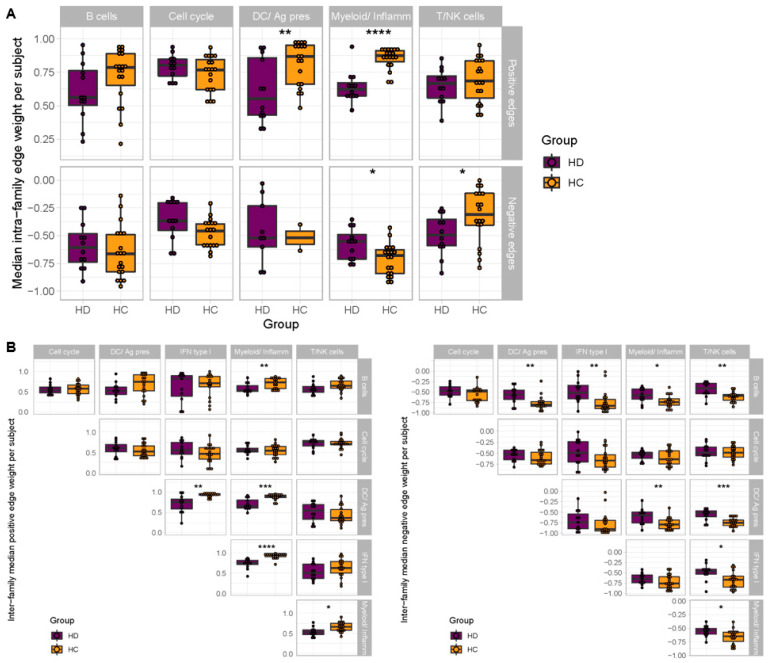
Comparison of single-subject BTM family associations between HC and HD groups show weakened positive and negative co-expression in HD subjects. (**A**) Box-and-whisker plots of median edge strength of intrafamily edges are shown for HD (purple) and HC (yellow) groups, separately for positive edges (top) and negative edges (bottom) for BTMs within each family (B cells, cell-cycle, DC/Ag pres, Myeloid/Inflamm, T/NK cells). (**B**) Median edge strength of interfamily edges is shown for HD and HC groups, separately for positive edges (left) and negative edges (right), pairwise between each family. * *p* < 0.05, ** *p* < 0.01, *** *p* < 0.001, **** *p* < 0.0001.

When comparing the median edge weights between HD and HC subjects at the single-subject level, the DC/APC and Myeloid/Inflamm families demonstrated significantly weaker positive intrafamily co-expression in HD subjects (*p* < 0.01, *p* < 0.0001), while the T/NK family was the only family to demonstrate stronger negative intrafamily co-expression in the HD group (*p* < 0.05) (Figure 4A). Additionally, the DC/APC family demonstrated weaker positive interfamily co-expression in the HD compared to the HC group (*p* < 0.05), including the weakening of positive interfamily regulation pairwise between the DC/APC, IFN type I, and Myeloid/Inflamm families (Figure 4B). The DC/APC family also showed weaker negative interfamily co-expression (*p* < 0.01) with the B cell, Myeloid/Inflamm, and T/NK BTM families (Figure 4B). The weakened negative co-expression with the T/NK family was the most significantly altered negative edge between all pairs of BTM families (*p* < 0.001), with the most weakened negative edge between the DC/APC BTM LI.M43.0 (Myeloid, dendritic cell activation via NfkB (I)) and T/NK BTM LI.M7.0 (enriched in T cells (I)). Both the weakened positive and negative edges were also seen in the group-level BTM co-expression networks (Figure 3). Taken together, the mRNA vaccine-induced immune response in HD subjects shows primary weakening of positive coregulation among DC/APC BTMs that is associated with the weaker positive coregulation of BTMs involved in innate immunity and the weaker negative coregulation of BTMs involved in cellular immunity, implicating the disruption of immune programming at the transcriptional level. 

### 2.5. Gene Regulatory Networks Reveal Weakened Transcriptional Regulation of Innate Immune Targets in HD Subjects

We investigated the precise transcriptional elements that may be driving the dysfunctional coregulation in HD subjects by constructing gene regulatory networks for each BTM using PANDA (Passing Messages between Networks for Data Assimilation) [21]. PANDA is a gene regulatory network reconstruction method that predicts regulatory relationships between gene expression and transcription factors using protein–protein interaction, gene expression, and sequence motif data. Our PANDA analyses demonstrated significant regulatory network differences for 35 BTMs (*p* < 0.05, FDR-adjusted). Of these BTMs, 25 exhibited weakened targeting in HD subjects and 10 exhibited stronger targeting. The top three most differentially targeted BTMs were LI.M161.0 (enriched in NK cells (II)), LI.M43.0 (myeloid, dendritic cell activation via NFkB), and LI.M7.2 (growth factor induced, enriched in nuclear receptor subfamily), all of which exhibited weakened regulation in the HD group. 

**Figure 5:**
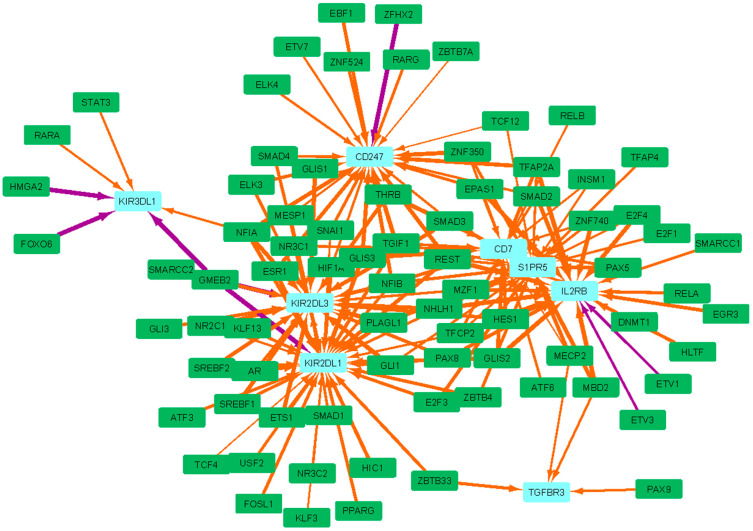
The LI.M61.0 (NK cells (II)) BTM is the most significantly dysregulated BTM in HD subjects. The top 150 most dysregulated edges involving the core enrichment genes, which contribute significantly to this enrichment of this BTM, are shown. Green rectangles are transcription factors (TFs), light blue rectangles are core enriched genes, orange edges are more weakly regulated in HD compared to HC subjects, and purple edges are more strongly regulated in HD compared to HC subjects.

In LI.M61.0 (enriched in NK cells (II)), the core enrichment genes consist of cell surface receptors on T cells and NK cells. In order of significance, these were TGFBR3, KIR2DS4, CD7, IL2RB, S1PR5, KIR2DL3, KIR3DL1, CD247, and KIR2DL1. The top 150 most dysregulated edges involving these core enrichment genes are shown in Figure 5. TGFBR3 encodes Type III TGF-beta receptor, which is a central co-receptor for the TGF-beta family required for high affinity binding, but can also undergo ectodomain shedding, ultimately inhibiting downstream signaling [22,23]. In fact, blocking the receptor has been shown to promote the TGFβ-dependent induction of Tregs [24]. The gene, therefore, plays a dual role in immune activation and tolerance. While most dysregulated gene targets in this BTM exhibit altered targeting primarily by transcription factors (TFs) that function both as activators and repressors, TGFBR3 demonstrates weaker targeting, predominantly by transcriptional repressors including MECP2 and MBD2, which bind methylated promoter regions of DNA [25,26]. Similarly, IL2RB, which is vital for T-cell-mediated immunity and immune tolerance via T regulatory cells (Tregs), [27] exhibited weakened regulation by many repressive TFs including MBD2, DNMT1, REST, E2F4, and SMAD2. Meanwhile, KIR2DS4, KIR2DL3, KIR3DL1, and KIR2DL1 encode NK cell receptors that interact with human leukocyte antigen class I molecules (HLA-I). While KIR2DS4 triggers NK cell degranulation upon binding to a conserved bacterial epitope of many human pathogens, KIR2DL3, KIR3DL1, and KIR2DL1 are inhibitory receptors [28,29]. The weakened targeting of LI.M61.0 can, thus, be broadly characterized as the dysregulation of both activating and tolerogenic receptors on T and NK cells.

**Figure 6:**
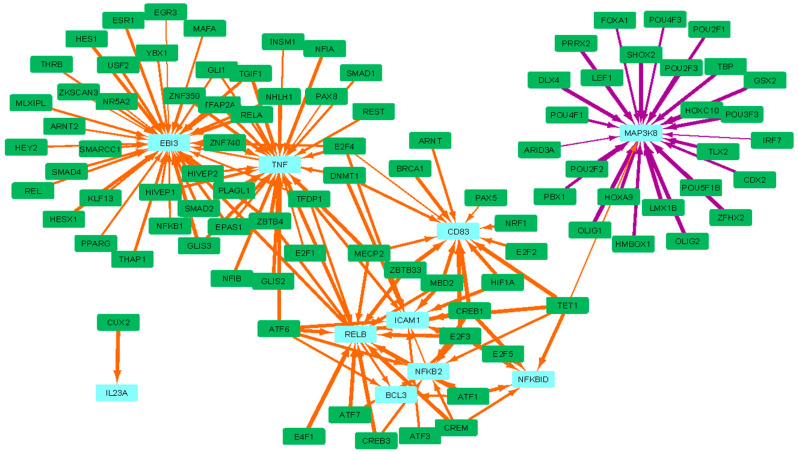
The LI.M43.0 (myeloid, dendritic cell activation via NFkB) BTM is the second most significantly dysregulated BTM in HD subjects. The top 150 most dysregulated edges involving the core enrichment genes, which contribute significantly to this enrichment of this BTM, are shown. Green rectangles are TFs, light blue rectangles are core enriched genes, orange edges are more weakly regulated in HD compared to HC subjects, and purple edges are more strongly regulated in HD compared to HC subjects.

The dysregulation of LI.M43.0 (myeloid, dendritic cell activation via NFkB (I)) is driven by the significantly altered targeting of core enrichment genes ICAM1, IL23A, NFKBID, VCAM1, EBI3, CD83, BCL3, RELB, TNF, NFKB2, and MAP3K8 (Figure 6). These genes represent various players in the NFkB pathway, including TNF, NFKB2, MAP3K8, BCL3, RELB, and NFkB inhibitor NFKBID, as well as cytokines and receptors expressed by DCs, including IL23A, EBI3, and CD83. Except for MAP3K8, which was more strongly regulated in HD patients, all of the remaining core enrichment genes exhibit decreased regulation by several transcriptional repressors, including the decreased targeting of ICAM by MBD2, DNMT1, and E2Fs, as well as the decreased targeting of TNF by ZBTB4, MECP2, TGIF1, ZNF350, and SMAD2.

**Figure 7:**
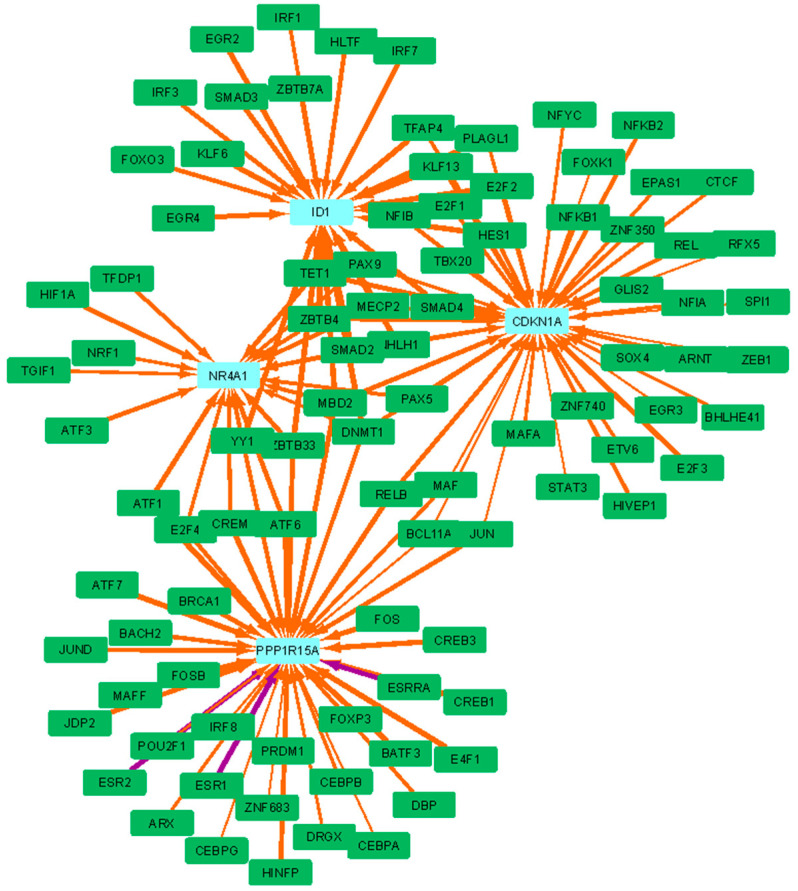
The LI.M94.0 (growth-factor-induced, enriched in nuclear receptor subfamily 4) BTM is the third most significantly dysregulated BTM in HD subjects. The top 150 most dysregulated edges involving the core enrichment genes, which contribute significantly to enrichment of this BTM, are shown. Green rectangles are TFs, light blue rectangles are core enriched genes, orange edges are more weakly regulated in HD compared to HC subjects, and purple edges are more strongly regulated in HD compared to HC subjects.

The dysregulation of LI.M94.0 (growth-factor-induced, enriched in nuclear receptor subfamily 4) is driven by the decreased regulation of NR4A1, PPP1R15A, ID1, and CDKN1A (Figure 7), of which the latter three are involved in apoptotic signaling [30,31,32]. The final core enriched gene is Inhibitor of Differentiation 1 (Id1), which is responsible for a switch from DC differentiation to myeloid-derived suppressor cell and Treg expansion, in response to TGF-beta [33]. All of these core enriched genes demonstrate decreased targeting by repressive transcription factors, including ZBTB33, MECEP2, MBD2, and DNMT1.

Notably, many of the enriched core genes in the BTMs with most weakened regulation in HD subjects were differentially targeted by MECP2 and MBD2, including TGFBR3, IL2RB, TNF, and all of the core enriched genes in LI.M94.0 (growth-factor-induced, enriched in nuclear receptor subfamily 4). MECP2 and MBD2 are members of a family of nuclear proteins with a methyl-CpG-binding domain (MBD), which typically binds to methylated promoters to repress transcription [25,26]. Interestingly, MBD2 was also shown to activate transcription by promoting demethylation in a Treg-specific demethylation region, resulting in Foxp3 expression and Treg suppressive function [34]. These insights suggest a potential role of altered DNA methylation of peripheral blood in the immune dysregulation of ESRD.

### 2.6. Deconvolution of Immune Cell Populations Reveals Persistent Discrepancies in Innate and Adaptive Immune Cell Types in HD Subjects

**Figure 8:**
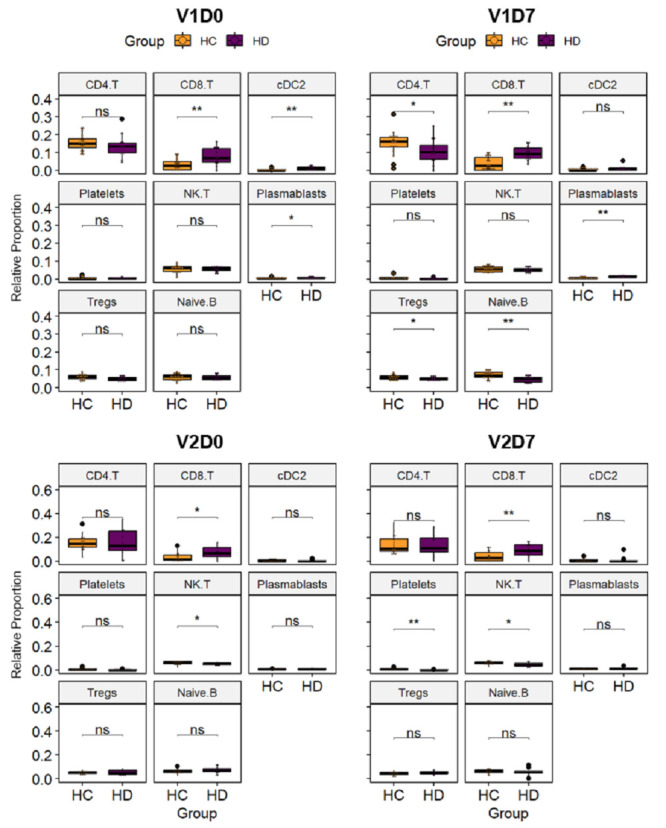
Cell deconvolution analysis of immune cell populations in HC and HD subjects show persistent discrepancies in innate and adaptive immune types over the course of the two-dose vaccination. Relative proportions of 8 significantly altered deconvoluted immune cell types are shown for HC (yellow) and HD (purple) subjects at four time points before and after vaccination with two doses (V1D0, V1D7, V2D0, V2D7). * *p* < 0.05, ** *p* < 0.01, ns = non-significant.

We next investigated how weakened coregulatory networks in HD subjects may be reflected in the affected immune cell populations. Cell deconvolution analysis provides a means to computationally identify cell populations from bulk RNA-seq expression data when such information would otherwise be inaccessible, though its accuracy is largely dependent on the reference signature markers used for distinguishing cell types [35]. Accordingly, the deconvolution of HC and HD cell populations across four time points (V1D0, V1D7, V2D0, V2D7) was performed based on a single-cell RNA-seq dataset derived from healthy PBMCs stimulated by the same SARS-CoV-2 mRNA vaccine (BNT162b2) and resolved at 16 immune cell types (CD4+ T, CD14+ monocytes, Naive CD8+ T, CD8+ T, CD16+ monocytes, NK, cDC2, B, pDC, HPCs, Platelets, NK T, Plasmablasts, Tregs, Naive B, cDC1) [36]. Overall, the relative proportions of most immune cells did not differ significantly between HD and HC samples across all time points, with half significantly differing between groups at at least one time point (Figure 8).

Differences in some cell types were observed between the HC and HD groups at baseline (V1D0), including higher proportions of CD8+ T, cDC2 cells, and plasmablasts in HD (*p* < 0.01, *p* < 0.01, *p* < 0.05). After stimulation with the first vaccine dose, cDC2 cells converged to similar proportions between the HD and HC groups by V1D7, but CD8+ T cells and plasmablasts remained significantly higher (*p* < 0.01, *p* < 0.01) while CD4+ T cells, Tregs, and naïve B cells were observed to be lower in HD subjects (*p* < 0.05, *p* < 0.05, *p* < 0.01). By V2D0, the difference in relative proportions of most of these immune cell types between the HD and HC subjects became non-significant, with CD8+ T cells still significantly increased in the HD group (*p* < 0.01) and platelets and NK T cells significantly decreased (*p* < 0.01, *p* < 0.05). Nonetheless, after stimulation with the second vaccine dose at V2D7, the relative proportion of platelets in the HD subjects became comparable to that in HCs as well (*p* < 0.05). While cDC1 proportions did not significantly change in HD subjects, weakened cDC1 function along with strong proinflammatory cDC2 activity had been reported in chronic inflammatory conditions [37]. Moreover, while cDC1s are primarily attributed to the activation of CD8+ T cells, [38] cDC2s have also been found to take on a hybrid role that includes CD8+ T cell priming during inflammation, [39] indicating that cDC2 may be driving the elevated baseline CD8+ T cells observed in the HD group. After vaccine stimulation, this discrepancy disappears as the cDC proportions in the HCs approach those of the inflammatory state in the HD subjects. More dynamic regulation of cDC2, as well as Treg and plasmablast, populations in HCs was also evident when comparing longitudinal changes pre- and post-vaccination with both doses (Figure S2). In contrast, naive B cells display a temporal delay in trending towards their peak for HD subjects, which explains the discrepancy observed at V1D7. 

It is important to note that, while the relative proportions of most immune cells appear similar between the HC and HD groups, we had previously reported that baseline WBC counts in HD subjects skewed toward the low end of normal limits [10]. This means that cell types with similar proportions between HCs and HD subjects at V1D0 may still translate to lower absolute counts in HD subjects. Altogether, these cell deconvolution findings illustrate a pre-existing difference in abundance of both innate and adaptive immune cell types in the HD group that persisted even after immune stimulation by the two-dose BNT162b2 mRNA vaccine. 

## 3. Discussion

Through BTM co-expression and gene regulatory network analyses, we illustrate how the delayed immune response to COVID-19 mRNA vaccination in the immunocompromised HD population is complicated by the defective coregulation of innate immune signaling. Our BTM co-expression network results demonstrate broadly weakened coupling between different components of the immune system in HD subjects compared to HCs, representing a global desensitization likely driven by chronic inflammation. In particular, BTMs in the DC/APC family exhibited the most weakened positive intrafamily coregulation. These network findings are further contextualized by our cell deconvolution analysis of conventional DC subtypes, in which a significantly higher proportion of cDC2s was noted with a concomitantly lower, albeit non-significant, proportion of cDC1s prior to vaccination in HD subjects compared to HCs. As a result, both the network and deconvolution results are consistent with evidence from the literature showing significantly decreased numbers of DCs in ESRD, which decline further with HD treatment [40], as well as the impaired maturation of monocytes and DCs and decreased antigen presentation [41,42,43].

The analysis of the BTM co-expression networks additionally showed weakened positive and negative interfamily coregulation between the DC/APC, IFN type I, and Myeloid/Inflamm families. DC dysfunction in ESRD has been proposed to stem from alterations in pattern recognition receptors (PRRs), including both the increased and decreased expression of TLR4 [13,44], increased expression of the secreted PRR mannose-binding lectin, and increased expression of major macrophage scavenger receptors SR-A and CD36 [14]. Interestingly, LI.M146 (MHC-TLR7-TLR8) was another BTM found to exhibit weakened targeting in HD subjects, and features the core enrichment genes TLR7 and TLR8, which have been shown to induce type 1 IFNs in DCs that synergize with the NFkB pathway to activate DCs [45]. Furthermore, the most significantly weakened edge in each of these interfamily relationships involving the DC/APC family was LI.M43.0 (Myeloid, dendritic cell activation via NFkB (I)), which is also the second most significantly dysregulated BTM from our PANDA network analysis, with core enrichment genes including NFkB pathway mediators such as TNF, NFkB2, and NFkBID. TNF, a proinflammatory cytokine that is upregulated by TLR binding and required for NFkB activation and DC maturation [46,47], is also required for both CD8+ T cell activation and apoptosis [48]. As effector CD8+ T cells possess the potential to differentiate into memory T cells, which can persist for as long as 10 years post-activation, decreased apoptosis may also lead to chronically elevated memory T cell populations [49]. As a result, its dysregulation of CD8+ T cell apoptosis may account for the chronically elevated CD8+ T cell populations seen in HD, as noted in this study as well as others [50,51]. Taken together, these results reinforce evidence of TLR dysfunction, with a mediating role of type 1 IFN and NFkB induction, leading to the impaired maturation and activation of DCs.

In addition to BTMs related to myeloid cells, the most significantly dysregulated BTM from our PANDA analysis was LI.M61.0 (enriched in NK cells (II)), with core enrichment genes comprising activating and tolerogenic receptors on T cells and NK cells. Though deconvoluted NK cell proportions did not significantly differ between HD and HC, there were significant reductions in CD4+ T cells at V1D7 and in NK T cells, which possess receptors characteristic of both NK and T cells [52] at V2D0 and V2D7 in HD relative to HCs. TGFBR3 and IL2RB are two core enriched genes that play a critical role in the balance of activation and tolerance via Tregs [24,27]. The weakened regulation of these genes may, thus, contribute to the disturbed Treg function reported in ESRD [53,54] as well as the significantly lower Treg proportions we observed in HD subjects at V1D7. In addition to Tregs, the IL-2 receptor is also involved in the differentiation of anti-inflammatory Th2 cells, which have been reported to be diminished in HD patients and may be reflected in the significantly lower CD4+ proportions observed in our HD subjects at V1D7.

Our regulatory network results from PANDA further identified regulators of cell survival and apoptosis in ESRD. The ESRD literature has demonstrated the accelerated apoptosis of neutrophils [55] as well as mixed findings of increased B cell apoptosis in one study [15], in contrast to increased B cell survival factors in another study [16]. Our results showed the weakened regulation of LI.M94.0 (growth-factor-induced, enriched in nuclear receptor subfamily 4), with differential targeting of three core enriched genes involved in apoptotic signaling (NR4A1, PPP1R15A, and CDKN1A). In conjunction, the deconvolution analysis of B cell subtypes in HD subjects revealed a significant decrease in naïve B cells relative to HCs’ proportions at V1D7, potentially as a result of increased apoptotic activity following vaccine-induced immune stimulation. In contrast, plasmablasts exhibited significantly higher proportions in HD than in HC subjects at V1D0 and V1D7. Immature B cells were shown to be elevated in another study on ESRD patients with HD treatment [56] and generally persist in this state in response to chronic stimulation [57]. Nonetheless, both naïve B cells and plasmablasts normalized to HC levels during V2D0 and V2D7 while mature B cell populations remained comparatively similar to HCs throughout the vaccine regimen, suggesting that the earlier observed discrepancies in B cell populations may be a product of delayed upstream signaling. This is consistent with the comparable antibody production and neutralizing function observed in mRNA-vaccinated ESRD patients relative to healthy controls, as previously demonstrated by our group and others [6,7,8,9,10]. The mixed findings of B cell apoptosis in ESRD are also likely to be context-dependent: Fernández-Fresnedo et al. cultured peripheral blood cells for four days prior to assessing apoptosis, while Pahl et al. assessed apoptosis on freshly isolated cells. 

There is a wealth of evidence demonstrating TLR-induced alterations of the epigenetic landscape, leading to both increased and decreased expression of TLR-induced genes [58]. For example, in macrophages, LPS signaling through TLR4 alters chromatin accessibility at TLR-responsive inflammatory genes including IL-6 [59]. In support of a mediating role of type 1 IFN in the TLR dysfunction leading to the impaired maturation and activation of DCs, type I IFN has also been shown to catalyze the methylation of promoters of NF-kB-responsive genes [60]. Additionally, oxidative stress has been shown to alter DNA methylation profiles, including in peripheral blood. In fact, oxidative damage to a methyl-CpG site in a methyl-binding protein recognition sequence has been shown to substantially reduce the binding affinity of MECP2 [61]. It is reasonable that, in addition to altering the regulation between immune players, epigenetic mechanisms could independently increase the susceptibility of immune subsets to apoptosis.

Our study is limited by its small sample size due to the selection of patients with samples collected across all time points. However, this is partly mitigated by allowing paired analysis across the time points [62]. Significant differences in mean age, racial/ethnic background, and past medical history commonly associated with ESRD between the HD and HC groups could additionally influence the differences observed between the two. Specifically, others have demonstrated weaker antibody immune responses in patients over 50 years old versus patients younger than 50 years old to the BNT162b2 vaccine [63]. While the insights made discernible through our bioinformatics approaches have basis in, and additionally expand on, findings from previous studies, they could be further validated through more direct measurements of the epigenetic and cellular changes in ESRD PBMCs using approaches such as ATAC-seq, DNA methylation profiling, and flow cytometry. 

Overall, we elucidated a complex regulatory interplay in ESRD with HD resulting in the simultaneous dampening of immune activation and tolerogenic immune responses that can be appreciated on both a group- and single-subject level. Moreover, by integrating our findings at the epigenetic, transcriptional, and cellular biological levels, we gained a more holistic view of which aspects of the immune system are perturbed and how dysregulation at one level may affect downstream immune cell populations and activity. Our results reinforce prior proposals that TLR dysfunction leads to the impaired maturation and activation of DCs in the HD population. Constitutive stimulation of TLRs may lead to low-grade baseline inflammation, simultaneously resulting in desensitization that impairs the ability of the immune system to mount immunogenic responses. While this may have stymied successful seroconversion by traditional vaccine modalities, mRNA vaccines may still mount robust, if delayed, antibody responses in HD patients comparable to their immunocompetent counterparts by stimulating both the innate and adaptive branches of the immune response. mRNA vaccines thus present a promising avenue to promote the immune-responsive vaccination of ESRD and HD patients against COVID-19 and other high-risk infections.

## 4. Materials and Methods

### 4.1. Cohort Recruitment and Sample Acquisition

The study was approved by the University of Illinois at Chicago IRB (#2018-1038) Ethics Review Committee. All research was performed in accordance with relevant regulations, and informed consent was obtained from all participants. Our recruited cohort and sample protocols for this study are the same as those previously described [10].

### 4.2. RNA Extraction and Sequencing

Our RNA extraction and sequencing (RNA-seq) protocols for this study are the same as those previously described [10].

### 4.3. Differential Gene Expression Analysis

Our bioinformatics approach to analyzing differential gene expression for this study are the same as those previously described [10].

### 4.4. Blood Transcription Module Enrichment Analysis

Gene set enrichment analysis was performed using blood transcription module (BTM) gene sets according to the same protocols as previously described [10]. To summarize these analyses, BTMs were categorized into different families reflecting immune cell types or immunologic processes (as characterized by Braun et al.): B cells, cell cycle, dendritic cell/antigen presentation, type I interferon (IFN type I), myeloid activity/inflammation, T/NK cells, and others [20]. The percentage of BTMs in each significantly enriched BTM family was then quantified at each time point.

### 4.5. Group-Level and Single-Subject Blood Transcription Module Network Construction

For BTM network construction, BTMs that demonstrated a significant effect of time point on eigengene expression were selected as candidate BTM nodes. The significance of time point was assessed using an ANOVA with the main effects of group and time point and the random effect of subject. The *p*-values for the main effect of time point were FDR-corrected across BTMs. Candidate BTMs with significant membership gene overlap were excluded by the following criterion: if candidate BTMs overlapped with a Jaccard index greater than 0.2, then only the BTM with the larger number of membership genes was retained. Using this final set of BTMs, pairwise Pearson correlations were performed between all BTM eigengenes across subjects and V1D0, V1D7, V2D0, and V2D7 samples, separately for each subject group to generate one group HC co-expression network and one HD co-expression network.

Single-subject co-expression networks were constructed in a similar fashion to the group networks, but with only four samples per network (V1D0, V1D7, V2D0, V2D7). Specifically, for each subject, a co-expression network was constructed using the same set of BTMs utilized in the HC and HD group co-expression networks. For a given subject, pairwise Pearson correlations were performed between all BTM eigengenes across all four time points.

### 4.6. Group-Level Blood Transcription Module Co-Expression Network Comparison

To compare the HC network to the HD network, Fisher’s Z transformation was applied to each network’s edges, and then the Z-transformed HC network was subtracted from the Z-transformed HD network to obtain a Z-score difference network. *p*-values for the Z-score difference network, calculated from a Z-score to *p*-value transformation, were FDR-adjusted across edges. Edges that were not significantly different between HD and HC after FDR correction were set to 0 in the Z-score difference network.

In order to characterize the level of coregulation within each given BTM family (and the difference between HC and HD subjects), intrafamily co-expression was investigated. Intrafamily co-expression represents a subnetwork in which nodes comprise all BTMs from a given family (e.g., B cells, cell cycle). For each BTM family subnetwork, we quantified the number of differentially co-expressed edges from the Z-difference network that were (1) positively co-expressed in both HD and HC subjects, but weaker (less positive) in HD, (2) positively co-expressed in both HD and HC subjects, but stronger in HD, (3) negatively co-expressed in both HD and HC subjects, but weaker (less negative) in HD, (4) negatively co-expressed in both HD and HC subjects, but stronger in HD, (5) positively co-expressed in HD subjects, but negative in HCs, (6) negatively co-expressed in HD subjects, but positive in HCs.

These numbers of dysregulated edges were then divided by the total number of possible edges within the BTM family (n choose 2, where n is the number of nodes in the BTM family), yielding the percentage of edges within each family demonstrating each class of differential co-expression. A similar approach was used to characterize the differential co-expression of BTMs between BTM families (interfamily co-expression). For each BTM family, percentages of dysregulated edges between BTMs within a given a family (first node) and BTMs outside of the family (second node) were quantified. Finally, percentages of dysregulated edges were quantified pairwise between the BTM families.

### 4.7. Single Subject-Level BTM Co-Expression Network Comparison

Edge weight (Fisher Z) distributions for single-subject co-expression networks were compared between HD and HC groups both globally and for each BTM family. A global statistical comparison of edge weights was achieved by quantifying the median positive edge weight per subject, and then comparing these between the HD and HC groups using a Student’s *t*-test. Global median negative edge comparisons were performed in the same way.

To characterize the differential co-expression of intrafamily BTMs, the median positive edge weight across all edges within a BTM family was calculated on a per-subject basis. These median edge weights were then compared between the HD and HC groups using a Student’s *t*-test. The median intrafamily negative edge weight within each BTM was compared in the same fashion.

A similar approach was used to characterize the differential co-expression of interfamily BTMs. For each BTM family, the median positive edge weight across all edges between BTMs within a given family (first node) and BTMs outside of the family (second node) were quantified and then compared between the HD and HC groups. The median interfamily negative co-expression was compared in the same fashion.

### 4.8. Gene Regulatory Network Construction and Analysis

To more specifically characterize the regulatory interactions underlying altered co-expression networks, gene regulatory networks were constructed separately for HD and HC groups using PANDA (Passing Messages between Networks for Data Assimilation) [21]. For the inputs to the PANDA algorithm, we used (1) an initial TF–gene regulatory matrix derived from the network on the Glass et al. website (https://sites.google.com/a/channing.harvard.edu/kimberlyglass/tools/resources (accessed on 23 March 2022), which was based on the TFs present in (2) our previously variance-stabilized gene expression matrix, and (3) a protein–protein interaction matrix derived from the STRING database interaction scores between all TFs used in the initial TF–gene regulatory matrix. The output regulatory network for controls was then subtracted from the output HD regulatory network, yielding a regulatory difference network. Gene set enrichment analysis was then performed using the *clusterProfiler* package [64] with BTM gene sets and a list of gene targets ranked by most significant edge differences from the regulatory difference network. BTMs with FDR-adjusted *p* < 0.05 were considered significantly enriched. The core enrichment genes, representing those genes that contribute most to the enrichment signal of the BTM, were obtained for the most enriched BTMs. 

### 4.9. Cell Deconvolution Analysis

To determine whether the regulatory findings are driven more by changes in immune cell populations than activity, we performed cell deconvolution analysis on our previously variance-stabilized gene expression matrix at four time points (V1D0, V1D7, V2D0, V2D7) using *CIBERSORTx* [65]. For the deconvolution reference, we constructed a custom signature matrix based on a published single-cell RNA-seq dataset of vaccinated control PBMCs to yield relative proportions for 16 immune cell populations (“CD4 T”, “CD14+ monocytes”, “Naive CD8 T”, “CD8 T”, “CD16+ monocytes”, “NK”, “cDC2”, “B”, “pDC”, “HPCs”, “Platelets”, “NK T”, “Plasmablasts”, “Tregs”, “Naive B”, “cDC1”), which should be more representative of the cell markers and populations expected in our vaccinated cohorts [36]. Wilcoxon rank sum tests were performed to assess significant differences in relative proportions between the HD and HC groups for each cell type at each time point, as well as between time points for each cell type.

## Figures and Tables

**Table 1:** Demographic (gender, age, race/ethnicity) and clinical data (body mass index [BMI] and past medical history of diabetes, hypertension, and positive COVID-19 infection) are shown for hemodialysis and control groups, with *p* < 0.05 considered significantly different between the two groups.

	Hemodialysis	Control	*p*-Value
Total Number of Subjects	12	19	
Gender			
Male	7	7	0.4233
Female	5	12	0.4233
Age	59 ± 12	46 ± 16	0.0224
Race/Ethnicity			
Black/African American	7	2	0.01428
Asian/Pacific Islander	1	2	1.0
White/Caucasian	2	10	0.1044
Hispanic/Latinx	2	5	0.8533
BMI (kg/m^2^)	25 ± 8	29 ± 8	0.1856
Medical History			
Diabetes	7	2	0.01428
Hypertension	8	3	0.01247
Positive COVID-19	6	4	0.1988

## Data Availability

The original RNA-seq data presented in the study are openly available in Gene Expression Omnibus (GEO) under the accession code GSE209985.

## Supplementary Materials

The following supporting information can be downloaded at https://www.mdpi.com/article/10.3390/vaccines12101146/s1: Figure S1. Overview of study design and analytical workflow. Peripheral blood mononuclear cells (PBMCs) were collected from healthy controls (HCs) and ESRD patients on hemodialysis (HD) at four time points before and after receiving two mRNA vaccine doses (V1D0, V1D7, V2D0, V2D7). After extraction, RNA was analyzed through RNA-seq, differential gene expression analysis, and gene set enrichment to identify key immune modules called blood transcription modules (BTMs) and their collective families. Co-expression networks were constructed from BTMs at the group and single-subject levels, and the degree of BTM co-expression was compared within each BTM family (intrafamily) and between families (interfamily). Gene regulatory networks were constructed from gene expression, transcription factor (TF)–gene regulatory interactions, and protein–protein interactions to illustrate predicted TF regulatory dynamics of core genes within each BTM. Cell deconvolution of gene expression data referenced vaccinated PMBC signatures to identify immune cell populations to support our network findings. Figure S2. Cell deconvolution analysis of immune cell populations in HC and HD subjects show alterations in innate and adaptive immune types over the course of two-dose vaccination. Relative proportions of four deconvoluted immune cell types (cDC2, Tregs, naïve B, and plasmablasts) were significantly altered over four time points before and after vaccination with two doses (V1D0, V1D7, V2D0, V2D7) in HC (yellow) and HD (purple) subjects. * *p* < 0.05, ** *p* < 0.01, *** *p* < 0.001, ns = non-significant.
