# Supplementary material for: Network Analysis of Dysregulated Immune Response to COVID-19 mRNA Vaccination in Hemodialysis Patients
Source: Vaccines (Basel). 2024 Oct 7;12(10):1146. doi: 10.3390/vaccines12101146 (PMC11511558; doi:10.3390/vaccines12101146)
Supplement: Supplementary file 1 [file vaccines-12-01146-s001.zip › vaccines-3162151-supplementary.pdf]

Supplementary Materials

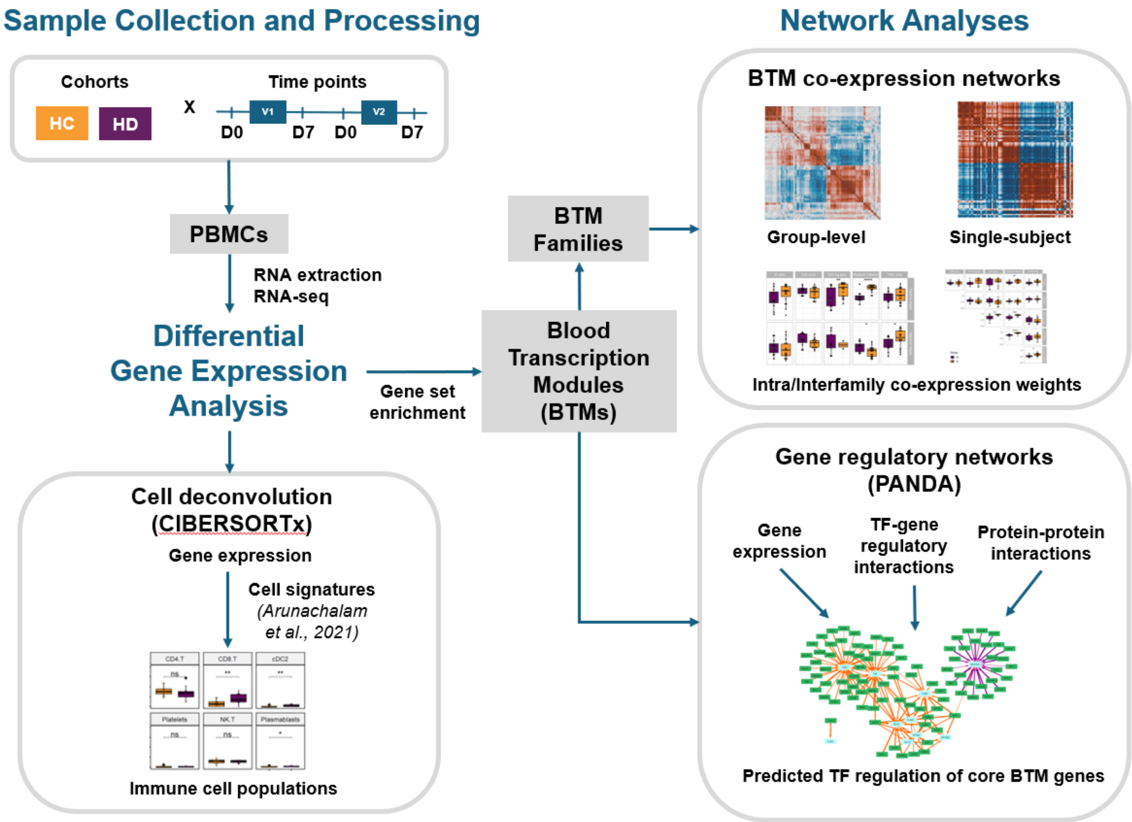

Figure S1. Overview of study design and analytical workflow. Peripheral blood mononuclear cells (PBMCs) were collected from healthy controls (HC) and ESRD patients on hemodialysis (HD) at four time points before and after receiving two mRNA vaccine doses (V1D0, V1D7, V2D0, V2D7). After extraction, RNA was analyzed through RNA-seq, differential gene expression analysis, and gene set enrichment to identify key immune modules called blood transcription modules (BTMs) and their collective families. Co-expression networks were constructed from BTMs at the group and single-subject levels, and the degree of BTM co-expression is compared within each BTM family (intrafamily) and between families (interfamily). Gene regulatory networks were constructed from gene expression, transcription factor (TF)-gene regulatory interactions, and protein-protein interactions to illustrate predicted TF regulatory dynamics of core genes within each BTM. Cell deconvolution of gene expression data referenced vaccinated PMBC signatures to identify immune cell populations to support our network findings.

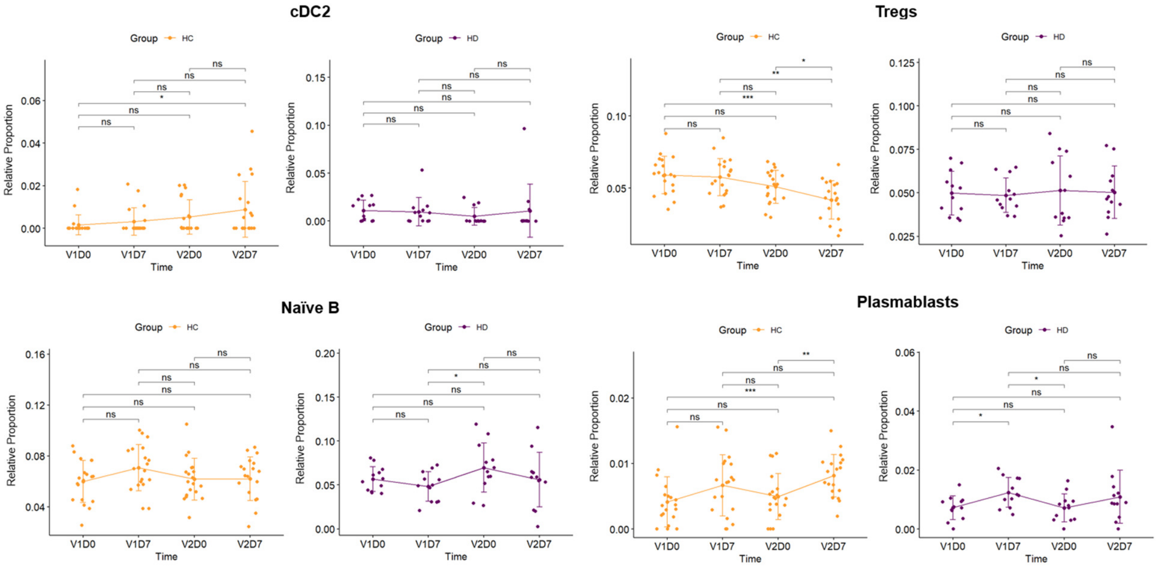

Figure S2. Cell deconvolution analysis of immune cell populations in HC and HD subjects show alterations in innate and adaptive immune types over the course of two-dose vaccination. Relative proportions of four deconvoluted immune cell types (cDC2, Tregs, naive B, and plasmablasts) were significantly altered over four time points before and after vaccination with two doses (V1D0, V1D7, V2D0, V2D7) in HC (yellow) and HD (purple). * $p < 0.05$, ** $p < 0.01$, *** $p < 0.001$, ns = non-significant.
